# Hepatitis B virus infection among medical aste handlers in Addis Ababa, Ethiopia

**DOI:** 10.1186/1756-0500-4-479

**Published:** 2011-11-03

**Authors:** Yitayal Shiferaw, Tamrat Abebe, Adane Mihret

**Affiliations:** 1 Department of Medical Laboratory Science, University of Gondar collage of Medicine & Health science, Gondar, PO.BOX:ET196, Ethiopia; 2 Department of Microbiology, Immunology& Parasitology, Addis Ababa University, Faculty of Medicine, Addis Ababa, PO.BOX:ET9086, Ethiopia; 3 Department of Microbiology, Immunology& Parasitology, Addis Ababa University, Faculty of Medicine, Addis Ababa, P.O.Box: ET 123 code1110, Ethiopia; 4 Armauer Hansen Research Institute, Addis Ababa, P.O.Box: ET 123 code1110, Ethiopia

## Abstract

**Background:**

Healthcare wastes contain a wide range of microorganisms among which hepatitis B virus (HBV) are the most significant pathogens. No data about the prevalence of HBV among medical waste handlers is available in Addis Ababa, Ethiopia. Therefore; this study was conducted to describe the prevalence of HBV infection among medical waste handlers in Government hospitals of Addis Ababa, Ethiopia.

**Findings:**

A cross sectional study was conducted among 252 medical and non-medical waste handlers working in three Government hospitals of Addis Ababa between May to July, 2010. Predesigned and tested questionnaire was used to collect soiociodemographic information. Blood sample was taken from 252 waste handlers and serum was tested for Hepatitis B surface antigen (HBsAg) and anti-Hepatitis core antigen (anti-HBcAg) using Enzyme Linked Immuno Sorbent Assay.

Of the 126 Medical Waste Handlers and 126 Non Medical Waste Handler, HBsAg was detected in 8 (6.3%) and 1 (0.8%), and anti-HBcAg in 60 (47.6%) and 40 (31.7%), respectively. Significant differences were observed in the detection rates of HBsAg (OR: 8, 95% CI: 1.02, 63.02; *p *= 0.01), Anti-HB c Ag (OR: 1.5, 95% CI: 1.1, 2.1; *p *= 0.01) and either markers (OR: 1.7, 95% CI: 1.2, 2.2; *p *= 0.001) in medical waste handlers compared to non medical waste handlers. 19.8% were trained to handle medical waste and none was immunized against HBV.

**Conclusion:**

This study shows a high prevalence of HBV infection in medical waste handlers compared to non medical waste handlers. Lack of training on how to handle medical waste among medical waste handlers was high.

## Background

Maintaining a clean environment and proper disposal of medical waste are social obligations of hospitals [1]. Exposure of patients, staff, waste handlers and the community to unnecessary health risks commonly occurs as a result of poor medical waste management practices in developing countries [2,3]. Due to their prevalence, and the severity of the infections they cause, hepatitis B virus (HBV), hepatitis C virus (HCV) and human immunodeficiency virus (HIV) account for most cases of occupational infection described in the literature [4].

HBV infection is one of the major diseases that cause serious public health problems. About 2 billion people globally have been infected, of which more than 350 million have chronic HBV. It has been estimated that, annually, about 1.2 million people die globally from chronic HBV infection, cirrhosis and liver cancer [5]. Global HBV endemicity ranges from high (≥ 8%) to intermediate (2-7%) and low (< 2%) [6]. In Addis Ababa, HBV prevalence (past or recent infection) was estimated to lie somewhere between 45% and 53%, with Hepatitis B surface Antigen (HBsAg) prevalence of 7% and a steady rise in seroprevalence (any marker) throughout childhood was observed, continuing in adult hood, rising to 70-80% in those aged 45-49 years [7].

Worldwide information on the spread of infection resulting from waste handling is limited. Studies from developed countries have shown that occupational exposure to waste may result in HBV infection [8]. Recently a study from the UK reported 40 incidents of sharps injuries associated with waste handling [9]. In Africa, some investigators accept 5% of all HIV infections are due to unsafe injections of which unsafe waste disposal is a component [10].

However, no data about the prevalence of hepatitis B virus among medical waste handlers is available in Ethiopia. Therefore; this study was conducted to compare the rate of HBV infection among medical waste handlers (MWHs) and non-medical west handlers (NMWHs) in Addis Ababa, Ethiopia. Association of age, gender, marital status, education level, HBV immunization, knowledge of HBV, their mode of transmission and previous training (on how to handle medical waste) with prevalence of this virus in MWHs were investigated.

## Materials and methods

### Study area &design

A cross-sectional study was conducted between May to July, 2010 in three government hospitals in Addis Ababa, Ethiopia (i.e., Tikur Anbessa General Specialized Hospital, Zeweditu Memorial and Yekatit 12 Hospital). These hospitals are among the largest hospitals in Ethiopia with a relatively advanced health establishments providing good quality medical care and medical education and training for medical and para-medical staff with a capacity of more than 600 beds. The nature and type of medical waste released from hospitals in Addis Ababa vary depending on their capacities and specialization. The studied hospitals generate all types of medical wastes.

### Study Population

There were 253 MWHs in three hospitals (141 in Tikur Anbessa General Specialized Hospital, 80 in Yekatit 12 Hospital and 32 in Zeweditu Memorial Hospital) who work in special working sites (i.e., laboratory, surgical, laundry, delivery and disposal sectors) and those in the service of in-patients and emergency wards.

### Sample size and Sampling technique

Since there was no particular study conducted in HBV prevalence among MWHs in Ethiopia, the sample size for our study was estimated using average prevalence of HBV (9%) obtained from similar studies in other parts of the world [11-14]. Single proportion formula with 95% confidence level (Z (1-ά/2) = 1.96) and 0.05 marginal errors was used to calculate 126 MWHs for this study. The sample size was distributed into three hospitals in a manner that resulted selection of equal proportion of MWHs from the total MWHs available in each hospital.

The list of 253 MWHs were obtained from Head's attendance sheet and 126 MWHs were selected using proportional stratified systematic sampling techniques taking in consideration of the variability in amount of MWHs in the three hospitals. In brief, 141 MWHs who were working in Tikur Anbessa General Specialized Hospital were stratified into different work sites and proportional number were selected using systematic random sampling technique that result a total of 70 MWHs (49.6%) from the total. Applying the same procedure 40 (50%) and 16 (50%) MWHs were selected from total MWHs working in Yekatit 12 (80 MWHs) and Zeweditu Memorial Hospitals (32 MWHs), respectively. Non-medical waste handlers (126) who were not directly or indirectly in contact with medical waste in the three hospitals were also enrolled to show the impact of type of waste handled as a risk for HBV infection.

#### Inclusion criteria

Non-vaccinated for HBV, volunteer.

#### Exclusion criteria

Vaccinated for HBV, non-volunteer, people under HBV treatment before working as medical waste handlers.

### Data collection

#### General information

General information about study participants was obtained out using a pre-tested self-administered questionnaire, which had four parts: (1) socio-demographic characteristics, history of immunization with Hepatitis B vaccine and other information; (2) the knowledge regarding blood borne infection including HIV, Hepatitis B and Hepatitis C, and their understanding of universal precautions;(3) occurrence of occupational exposure to blood and body fluid in mucous membrane during their work in the last twelve months; (4) occurrence of percutaneous injury (PCI) and responses regarding exposures and behaviors.

#### Blood sample

Blood sample was obtained from 126 MWHs and 126 NMWHs. A standard procedure was used to collect blood and process them for testing. All sera were screened for hepatitis B surface antigen (HBsAg) and antibody to hepatitis B core antigen (anti-HBcAg) using Enzyme Linked Immunosorbent Assay (ELISA) (Hepanostika test kit; Biomerieux, Boxtel, The Netherlands) in regional laboratory which is found in Zeweditu Memorial Hospital compound. Sera from each laboratory transported in cold box to examination site in the same day of collection.

#### Quality control

To insure the quality of laboratory results, the standard operating procedures of the company was followed every time and all tests were performed by experienced medical laboratory technologist. Furthermore known positive and negative control sera provided by the manufacture of the kits were used for every panel of the test.

#### Data analysis

Data were analyzed using SPSS V.16 software. Differences in proportions were evaluated by Pearson's χ^2 ^test; p < 0.05 was considered to be statistically significant. Bivariate and multivariate logistic regression analyses were used to assess the crude and adjusted effect of seemingly significant predictors of the target outcome. Odds ratio was used as a measure of the strength of association.

#### Ethical considerations

The study was approved by the institutional review board of Addis Ababa University Medical Faculty. Permission to conduct the study were obtained from the Director of each Hospitals and Lead Medical waste handlers. All the Medical waste handlers were given verbal information about the study and were assured about the confidentiality, protection and anonymity of data. Informed consent was obtained in writing from the individuals participating voluntarily. Positive individuals were referred to hospital's physicians for management.

## Results

From the total of 253 medical waste handlers in the three hospitals, 126 MWHs (49.8% of the study population) were participated for interview and gave blood samples. Complete information was obtained from all study participants and included in analysis. Some of the sociodemographic characteristics of waste handlers are shown in Table 1. The age range of the participants was 20 to 57 years (mean, standard deviation [SD] 35.7 [9.7] years) for MWHs and 21 to52 years (mean, standard deviation [SD] 33.5 [6.7] years) for NMWHs. The majority of the medical waste handlers were female (female to male ratio = 2.15:1) and 59.3% had secondary or more school education. The mean length of employment was 12 years, and 30.2% work in in-patient wards and 6.3% work in laboratory (Table 1).

**Table 1:** Sociodemographic Characteristics of Medical and Non Medical waste Handlers.

Socio demographic characteristic	MWHs	NMWHs
	NO (%)	NO (%)
Gender		
Male	40(31.7)	35(27.8)
Female	86(68.3)	91(72.2)
Age		
Mean ± SD	35.7 ± 9.7	33.5 ± 6.7
*Length of Service*		
*Median*	9.5	7.5
*mean+ *SD	12 ± 10	10 ± 6.5
*Educational status*		
*Primary or Less*	58(46)	55(43.7)
*Secondary or More *	68(54)	71(56.3)
*Marital Status*		
*single*	28(22.2)	33(26.2)
*Married *	98(77.8)	93(73.8)
Work site		
In patient wards	38(30.2)	
Emergency wards	18(14.3)	
Surgical site	20(15.9)	
Laboratory	8 (6.3)	
Delivery room	12(9.5)	
Laundry	15(11.9)	
Disposal sectors	15(11.9)	
Cafeterias		20(15.9)
Students & residence staffs dormitories		21(16.7)
Administration offices		40(31.7)
Class rooms		45(35.7)

From 126 MWHs and 126 NMWHs, HBsAg was detected in 8 (6.3%) and 1 (0.8%), and Anti-HBcAg in 60 (47.6%) and 40 (31.7%) respectively. Significant differences were observed in the detection rates of HBs Ag (OR: 8, 95% CI: 1.02, 63.02; *p *= 0.01), anti-HB c Ag (OR: 1.5, 95% CI: 1.1,2.1; *p *= 0.01) and either markers (OR: 1.7, 95% CI: 1.2,2.2; *p *= 0.001) in MWHs when compared with NMWHs (Table 2).

**Table 2:** Distribution of medical waste handlers and non-medical waste handlers according to hepatitis B virus markers.

Hepatitis B virus markers	No.(%) positive		OR(95%CI)	P- value
			
	MWHs(N = 126)	NMWHs(N = 126)		
HBsAg	8(6.3)	1(0.8)	8(1.02,63.02)	0.01
Ant-HBc	60(47.6)	40(31.7)	1.5(1.1,2.1)	0.01
Either markers	68(54)	41(32.5)	1.7(1.2,2.2)	0.001

Bivariate analysis of HBV prevalence in MWHs indicated more percentage of HBsAg was identified in male (7.5%), in single (14.3%) and age group between 20-29 years (10.3%). However; none of them showed statistical significant association. In contrast to HBsAg, significant amount of anti-HBcAg detected in male (OR: 1.6, 95% CI: 1.2, 2.3; *p *= 0.007) and age group between 40-49 years (OR: 10, 95% CI: 3.7, 32.1; *p *= 0.001) (Table 3).

**Table 3:** Distribution of HBsAg and anti-HBc in relation to Sociodemographic characteristics of Medical waste Handlers.

Socio demographic characteristic	N (%)	HBsAg in MWHs (N = 126)			Anti-HBc in MWHs (N = 126)		
		
		n (%)	OR (95% CI)	p-value	n (%)	OR (95% CI)	p-value
Gender							
Male	40(31.7)	3 (7.5)	1.3 (0.32,5.1)	0.72	26(65)	1.6(1.2,2.3)	0.007
Female	86(68.3)	5 (5.8)			34(39.5)		
Age				0.82			0.001
20-29	39 (31)	4 (10.3)	1		11 (28.2)	1	
30-39	38(30.1)	2 (5.3)	0 .49(.08,2.8)	0.42	16 (42.1)	1.9(0.72,4.9)	0.2
40-49	37(29.4)	2 (5.4)	0.5(0.09,2.9)	0.44	31 (83.8)	10 (3.7,32.1)	0.001
50-59	12(9.5)	0 (0)	UD	0.99	2 (16.7)	0.5(0.1,2.7)	0.43
Length of Service(years)				0.43			0.3
0-5	36(28.6)	1(2.8)	1		13(36.1)	1	
6-10	33(26.2)	1(3)	1.1(0.07,18.2)	0.95	15(45.5)	1.5(0.56,3.9)	0.43
11-15	31(24.6)	3(9.7)	3.8(.37,38)	0.26	18(58.1)	2.5(0.92,6.6)	0.08
> 15	26(20.6)	3(11.5)	4.6(.45,,46.6)	0.2	14(53.8)	2 (0.74,5.7)	0.17
*Educational status*							
*Primary or Less*	58 (46)	3 (5.2)	0.8 (0.16,2.3)	0.72	28 (48.3)	0.95(0.47,1.9)	0.89
*Secondary or More*	68 (54)	5 (7.4)			32 (47.1)		
*Marital Status*							
*Single(2**8**)*	28(22.2)	4 (14.3)	2.4 (0.52,10.4)	0.28	12 (42.9)	0.88(0.55,1.4)	0.57
*Married(98)*	98(77.8)	4 (4.1)			48(49)		

N = Total number of individuals in each category

n = Total number of positive individuals in each category

UD = Undefined

Likewise, a higher proportion of anti-HBcAg positivity was observed in those with a history of blood and body fluid splash in the eye, nose and mouth (48.6%) followed by those with history of blunt -penetrating injuries (19.8%). However; none of the studied risk factors was associated with ant-HBcAg positivity (Table 4).

**Table 4:** Distribution of anti-HBc Ag positivity among medical waste handlers with potential risk factors.

Risk factors	No. (%)	No. Anti-HBc positive (%)	OR (95% CI)	P-value
Liver disease	2(1.6)	0(0)	UD	0.17
Blood transfusion	19(15.1)	0(0)	UD	0.99
Body fluid splash in MM	35(27.8)	17(48.6)	0.95 (0.63,1.4)	0.81
Tattooing	21(16.7)	2(9.5)	0.09 (0.02,0.39)	0.001
Blunt-penetrating injuries	25(19.8)	15(60)	1.9 (0.77,4.51)	0.17
Dental procedure	8(6.3)	1(12.5)	0.14 (0.02,1.2)	0.07
Surgical procedure	5(4)	1(20)	0.26 (0.03,2.4)	0.22
Catheterization	4(3.2)	1(25)	0.36 (0.04,3.5)	0.38
No risk factors	25(19.8)	23(92)	0.39(0.29,0.52)	0.001
Total	126	60(47.6)		

UD = Undefined

In multivariate analysis of selected variables for independent predictors of HBV in MWHs, none of them showed significant effects (Table 5).

**Table 5:** Multivariate analysis of selected independent variables with Anti-HBc

Independent variables	N (%)	Anti-HBc n (%)	B	S.E	p-value	Exp(B)	95% C.I.for EXP(B)	
							
							Lower	upper
age					.021			
30-39	38 (30.1)	16 (42.1)	20.9	1.54	.999	1.14	.000	
40-49	37 (29.4)	30 (83.8)	43.5	1.96	.998	7.65	.000	
50-59	12 (9.5)	2 (16.7)	40.1	1.96	.998	2.55	.000	
sex(male)	40 (31.7)	26 (65)	21.6	8.48	.998	2.38	.000	
Length of employment					.993			
6-10	33 (26.2)	15 (45.5)	0.28	1.73	1.000	1.33	.000	
11-15	31 (24.6)	18 (58.1)	0.36	2.45	1.000	1.44	.000	
> 15	26 (20.6)	14 (53.8)	0.65	2.45	1.000	1.92	.000	
Education (primary or less)	58 (46)	28 (48.3)	-20.5	1.21	.999	.000	.000	
Splash exposure(yes)	35 (27.8)	18 (51.4)	-3.3	2.35	1.000	.04	.000	
Tattooing (yes)	21 (16.7)	2 (9.5)	-18.4	8.03	.998	.000	.000	
Blunt-penetrating injuries (yes)	25 (19.8)	15 (60)	2.1	2.37	1.000	7.91	.000	
History of surgery (yes)	5 (4)	1 (20)	-17.6	6.64	.998	.000	.000	
No risk factors(yes)	25 (19.8)	24 (96)	37.5	1.08	.997	1.91	.000	

N = Total number of MWHs in each category

n = number positive for Anti-HBc

All of MWHs participated in the study had knowledge of Acquired Immune Deficiency Syndrome (AIDS) and 55.6% of them had knowledge of viral hepatitis. In addition, all of them were aware that medical waste contains harmful pathogens and possibility of virus transmission at the time of collection, transportation and disposal of medical wastes. None of the medical waste handlers were immunized for HBV and only 25 (19.8%) were trained how to handle medical waste. All MWHs accredited that wearing protective equipments is very important to protect them from infection. However, 52 (41.3%) of them were not using personal protective equipment (PPE) regularly.

From 52 medical waste handlers who did not wear PPE regularly, 43(82.7%) reasoned shortage of supply, 25 (48.1%) difficulty to work with it and 18 (34.6%) loss of comfort. All medical waste handlers wore overalls, 100 (79.4%) thick disposable gloves, 85 (67.5%) boots and 29 (23%) wore masks. Male MWHs were significantly more likely to wear Boots (OR: 1.505: *P *< 0.002) compared to Female (Table 6).

**Table 6:** Use of Personal Protective Equipment (PPE) among male & female medical waste handlers.

Type of PPE	male(N = 40)	Female(N = 86)	Total(N = 126)	0dds ratio	95% CI	p-value
**Thick disposable glove**	30(75)	70(81.4)	100(79.4)	0.92	0.7,1.1	0.4
**Boots**	35(87.5)	50(58.1)	85(67.5)	1.5	1.2,1.7	0.002
**Mask**	10(25)	19(22.1)	29(23)	1.1	0.6,2.2	0.7
**Head covers**	30(75)	80(93)	110(87.3)	0.83	0.7,0.97	0.4
**overalls**	40(100)	86(100)	126(100)			NS

NS-not significant

## Discussion

Data on infection rates for HBV among MWHs worldwide are scarce. In the present study we found 6.3% and 47.6% of MWHs had HBsAg and anti-HBcAg, respectively. This rate was significantly higher compared to NMWHs. Although direct comparison is difficult because of methodological and study population differences, the prevalence we report, (6.3% HBsAg, 47.6% anti-HBc), appeared to be comparable to a previous estimate of HBV infection rate in the voluntary counseling and testing (VCT) clients and known HIV-positive cases of St Paul's General Specialized Hospital, Addis Ababa, Ethiopia (4.7% HBsAg and44.8% anti-HBc) [15]. It is also consistent with the general population of Addis Ababa (7% HBsAg, 45-53% past and current HBV infection) [7].

Significantly higher prevalence of anti-HBcAg was observed in age group between 40-49 years compared to other age groups (OR: 10, 95% CI: 3.7, 32.1; *p *= 0.001). Similar results were reported in different studies [11,12,14,15]. This increase in the rate of anti-HBcAg positivity with age may be due to the increased risk of exposure to HBV infection with time as observed in this study that MWHs in older age groups worked as medical waste handler for many years compared to the younger groups (data not presented here).

Again the rates of HBsAg (non-significantly) and anti-HBcAg (significantly) were higher in male compared to female medical waste handlers. It is difficult to explain the higher rate of HBV infection in men as they had similar exposure to HBV risk factors as women. Of course, there may be differences in risk behavior by gender in the early years of life [7]. However, a study indicated that males when infected with HBV are more likely to become carriers of the virus and females are more likely to develop anti-HBsAg that can clear HBsAg from blood circulation [16]. This contributes for continual existence of HBsAg and anti-HBc carriers males compared to females.

None of the observed risk factors showed statistically significant association with HBV infection in this study. However; the rate of occurrence of body fluid exposure to mucous membranes and blunt-penetrating injuries in work sites were higher. The stated prevalence of blunt-penetrating injuries or splash of blood or body fluid might reflect the level of occupational safety practice in the studied hospitals being below the standard, as protective equipment and clothing were not available for most workers. Furthermore lack of training about how to handle medical wastes was reflected by the fact that only 19% were trained for short duration ending with poor skill that create difficulties to use even the available PPE properly to protect themselves from accidents.

On top of these, absence of well defined policies related to medical waste management and huge lack of medical waste management infrastructure can lead to wrong disposal of wastes as observed like disposal of sharps in overfilled safety boxes, in containers intended only for non-sharp items, manual collection and transport of sharps mixed with other solid and liquid medical wastes. These wrong deeds might create difficulties for MWHs and contributes for the increased occurrence of the observed accidents.

Studies on seroprevalence of HBV in healthcare workers have clearly demonstrated the effectiveness of HBV vaccine in preventing infections [17,18] yet, none of the MWHs was immunized. Compared to this study better HBV immunization coverage among MWHs were reported in Turkey (27.5%) [12], Libya (27.5%) [11] and UK (21%) [9] where they have been implemented free HBV immunization to risk groups. In Ethiopia universal infant HBV immunization started in 2007. However, there is no universal availability of the vaccine for adult population and this may be the reason for this gap.

## Limitations

The study was conducted in only three hospitals among 126 MWHs; therefore, its generalizablity is limited. When assessing the association between occupational exposure and risks for HBV infections, limitations of the cross-sectional study design have to be taken into consideration.

It appeared that there was no clear policy for workers vaccination against infective diseases, and there was no medical examination for workers either before or during employment. Therefore, it was difficult for us to know the observed infections in MWHs were occurred before or after employed as medical waste handlers and this prevent us to say something about cause and effect relationship.

## Conclusion

This study showed a high prevalence of HBV infection in medical waste handlers compared to non-medical waste handlers. Anti-HBc was significantly higher in male and age group between 40-49 years. Lack of immunization against HBV, training on how to handle medical waste and universal precaution application were observed among MWHs. Training, immunization and post-exposure protection of MWHs, in addition to proper management of medical waste by the health authorities, may significantly reduce the risk of HBV infections among MWHs of Ethiopia.

## Competing interests

The authors declare that they have no competing interests.

## Authors' contributions

AM and TA conceived the study. AM, TA and YS initiated and designed the study. YS conducted the laboratory work, undertook statistical analysis and drafted the manuscript. AM and TA corrected the manuscript. All authors contributed to the writing of the manuscript and approved the submitted version of the manuscript.
